# MiR-200a-3p protects against myocardial ischemia-reperfusion injury via KEAP1–NRF2 signaling

**DOI:** 10.3389/fphys.2026.1826306

**Published:** 2026-05-01

**Authors:** Yanbo Zhao, Lulu Liu, Xiaohua Shen, Min Wang, Meihui Wang, Lingling Sun, Kai Zhang

**Affiliations:** 1 Department of Cardiology, Sir Run Run Shaw Hospital, Zhejiang University School of Medicine, Hangzhou, Zhejiang, China; 2 Zhejiang Key Laboratory of Cardiovascular Intervention and Precision Medicine, Hangzhou, Zhejiang, China; 3 Engineering Research Center for Cardiovascular Innovative Devices of Zhejiang Province, Hangzhou, Zhejiang, China

**Keywords:** cardioprotection, Keap1–Nrf2 pathway, MiR-200a-3p, myocardial ischemia/reperfusion injury, oxidative stress

## Abstract

**Background:**

Myocardial ischemia/reperfusion (I/R) injury is a major challenge in reperfusion therapy for acute myocardial infarction, primarily due to excessive oxidative stress, inflammation, and cardiomyocyte apoptosis. MicroRNAs are known regulators of cellular stress responses, but the role and underlying mechanism of miR-200a-3p in myocardial I/R injury remain unclear.

**Methods:**

*In vitro*, hypoxia/reoxygenation (H/R) hypoxia/reoxygenation (H/R)-treated human AC16 cardiomyocytes were used to assess the effects of miR-200a-3p modulation on cell viability, apoptosis, oxidative stress, and inflammatory cytokines. The interaction with KEAP1 and downstream NRF2 activation was examined using luciferase assays and protein analyses. *In vivo*, cardiac-specific AAV9-mediated miR-200a-3p overexpression in mice subjected to I/R injury was evaluated for myocardial injury, oxidative stress, inflammation, apoptosis, and KEAP1–NRF2 signaling.

**Results:**

MiR-200a-3p was markedly downregulated in H/R-treated cardiomyocytes and in mouse hearts after I/R injury. Restoring miR-200a-3p enhanced cell viability, reduced apoptosis, ROS accumulation, lipid peroxidation, and inflammatory cytokine release, and restored antioxidant defenses *in vitro*. *In vivo*, cardiac-specific miR-200a-3p overexpression attenuated myocardial injury, oxidative stress, inflammation, and cardiomyocyte apoptosis. Mechanistically, miR-200a-3p directly targeted KEAP1, promoted NRF2 nuclear translocation, and upregulated downstream antioxidant enzymes including HO-1 and NQO1, with KEAP1 suppression required for its cardioprotective effects.

**Conclusion:**

These findings indicate that miR-200a-3p protects against myocardial I/R injury by targeting KEAP1 and activating NRF2-dependent antioxidant signaling, identifying a novel redox-regulatory axis with therapeutic potential, with beneficial effects on myocardial injury and its associated functional impairment.

## 1 Introduction

Myocardial ischemia/reperfusion (I/R) injury is an unavoidable complication of reperfusion therapy for acute myocardial infarction, leading to cardiomyocyte damage, apoptosis, and impaired cardiac function (Cai and Yang, 2025). Excessive generation of reactive oxygen species (ROS) during I/R directly damages cellular membranes, mitochondria, and proteins, and triggers apoptosis or ferroptosis, contributing to cardiac dysfunction (Kurian et al., 2016; Al-Awar and Hussain, 2024). Oxidative stress is known to activate multiple signaling pathways, thereby exacerbating myocardial injury (Kamboj et al., 2025). The severity of I/R injury is further influenced by metabolic factors, such as left ventricular wall motion abnormalities in diabetes and elevated plasma asymmetric dimethylarginine (ADMA) (Corradi et al., 2024; Davari et al., 2025). These observations underscore the contribution of oxidative stress and metabolic dysregulation to myocardial vulnerability, highlighting the need to explore novel cardioprotective mechanisms.

MicroRNAs (miRNAs) are small non-coding RNAs that regulate cardiovascular physiology by targeting specific mRNAs, thereby influencing cell proliferation, differentiation, apoptosis, and stress responses (Kong et al., 2022). They are critical modulators of redox homeostasis and inflammatory signaling, both of which play central roles in myocardial injury (Schütte et al., 2023; Mehanna et al., 2025). Among these, miR-200a-3p has been reported to attenuate oxidative stress and inflammation in non-cardiac systems, such as spinal cord injury (Liu et al., 2026) and breast cancer models (Eades et al., 2011). In cardiovascular contexts, previous studies have shown that miR-200a-3p can influence cardiomyocyte apoptosis and inflammatory responses (Sun and Zhang, 2019; Chen et al., 2021), and its expression is altered in myocardial I/R injury (Li et al., 2024). However, these studies mainly describe its phenotypic effects, and the precise upstream–downstream regulatory mechanisms through which miR-200a-3p modulates oxidative stress in cardiomyocytes remain insufficiently defined, particularly in relation to canonical antioxidant signaling pathways.

Kelch-like ECH-associated protein 1 (KEAP1) is a key negative regulator of cellular antioxidant defenses, promoting ubiquitination and proteasomal degradation of nuclear factor erythroid 2–related factor 2 (NRF2) under basal conditions (Peng et al., 2023). Upon oxidative stress, KEAP1 inhibition allows NRF2 to translocate into the nucleus, where it binds antioxidant response elements (AREs) and induces genes such as HO-1 and NQO1, thereby enhancing the cell’s capacity to neutralize ROS (Suzuki et al., 2013). The KEAP1–NRF2 axis is widely recognized as a key protective mechanism in myocardial I/R injury (Dodson et al., 2019). Notably, miR-200a has been shown to directly target KEAP1 and activate NRF2 signaling in several non-cardiac models, including osteoblasts (Zhao et al., 2017) and renal disease (Wei et al., 2014a). Despite these findings, whether miR-200a-3p directly regulates the KEAP1–NRF2 pathway in cardiomyocytes during I/R injury, and whether this interaction functionally contributes to cardioprotection *in vivo*, remains unclear.

Therefore, we hypothesize that miR-200a-3p is dysregulated during myocardial I/R injury and protects cardiomyocytes by negatively regulating KEAP1 to activate NRF2 signaling, thereby mitigating oxidative stress. This study aims to elucidate the functional role and underlying mechanism of the miR-200a-3p/KEAP1/NRF2 pathway in myocardial I/R injury, providing a molecular basis for potential targeted cardioprotective strategies.

## 2 Materials and methods

### 2.1 Cell culture and H/R injury model

Human AC16 cardiomyocyte−like cells (Cat. No. CRL−3568; ATCC, Manassas, VA, USA) were maintained in Dulbecco’s modified Eagle’s medium (DMEM; Gibco, USA) supplemented with 10% fetal bovine serum (FBS; Gibco), 1% penicillin–streptomycin (Gibco) at 37 °C in a humidified incubator with 5% CO_2_. To establish the *in vitro* hypoxia–reoxygenation (H/R) injury model, AC16 cells at ~70–80% confluence were transferred to a hypoxia incubator (1% O_2_, 5% CO_2_, balance N_2_) for 16 h, followed by 6 h of reoxygenation under normoxic conditions (21% O_2_, 5% CO_2_), as described previously (Cheng et al., 2025). Cells maintained continuously in a standard incubator (21% O_2_, 5% CO_2_) served as the control group. AC16 cells, although immortalized, retain key cardiomyocyte-like characteristics and are widely used for *in vitro* studies of myocardial ischemia/reperfusion injury and oxidative stress, particularly for mechanistic investigations due to their stable phenotype and suitability for genetic manipulation (Du et al., 2022; Li et al., 2023).

### 2.2 Cell transfection

MiR-200a-3p mimic, miR-200a-3p inhibitor, KEAP1-specific siRNA (si-KEAP1), KEAP1 overexpression plasmid (OE-KEAP1), and their respective negative controls (mimic NC, inhibitor NC, si-NC, and empty vector) were obtained from GenePharma (Shanghai, China). AC16 cells were transfected using Lipofectamine 3000 (Invitrogen, USA) following the manufacturer’s protocol. Forty-eight hours post-transfection, cells were subjected to H/R treatment for downstream analyses.

### 2.3 Dual-luciferase reporter assay

The putative binding site between miR-200a-3p and the KEAP1 3′ untranslated region (3′UTR) was first identified using the TargetScan database. Based on the predicted interaction, wild-type (WT) and corresponding mutant (MUT) fragments of the KEAP1 3′UTR were generated and inserted into the pmiR-GLO luciferase reporter vector (Promega, Madison, WI, USA). HEK293T cells were transiently co-transfected with the WT or MUT reporter constructs together with miR-200a-3p mimic or negative control mimic using Lipofectamine 2000 (Invitrogen). After 48 h, luciferase activities were measured using a dual-luciferase reporter assay kit (Promega). Firefly luciferase activity was normalized to Renilla luciferase activity, and the data were expressed as relative luciferase activity. All experiments were independently repeated at least three times.

### 2.4 Cell viability assay

Cell viability was evaluated using the Cell Counting Kit−8 (CCK−8; Dojindo, Japan). AC16 cells were seeded in 96−well plates at 5 × 10³ cells per well. Following the indicated treatments, 10 μL of CCK−8 reagent was added to each well and incubated at 37 °C for 2 h. Absorbance at 450 nm was measured with a microplate reader, and viability was calculated as a percentage relative to untreated control cells. All experiments were performed in triplicate.

### 2.5 Flow cytometry analysis of apoptosis

Cell apoptosis was quantified using an Annexin V−FITC/propidium iodide (PI) apoptosis detection kit (Thermo Fisher Scientific, USA). After the indicated treatments, AC16 cells were harvested, washed twice with cold phosphate-buffered saline (PBS), and resuspended in binding buffer at a density of 1 × 10^6^ cells/mL. Cells were then incubated with 5 μL Annexin V−FITC and 5 μL PI for 15 min at room temperature in the dark. Immediately after staining, apoptotic cells were analyzed using a FACSCalibur flow cytometer (BD Biosciences, San Jose, CA, USA). All assays were performed in triplicate.

### 2.6 Measurement of intracellular ROS

Intracellular reactive oxygen species (ROS) levels were determined using the cell-permeable fluorescent probe 2′,7′-dichlorofluorescin diacetate (DCFH-DA; Elabscience, Shanghai, China). AC16 cells from each treatment group were harvested and washed twice with PBS. Cells were then incubated with 10 μM DCFH-DA in serum-free DMEM for 30 min at 37 °C in the dark. After incubation, cells were washed three times with PBS, resuspended in PBS, and immediately analyzed using a FACSCalibur flow cytometer (BD Biosciences). Experiments were performed in triplicate.

### 2.7 Immunofluorescence staining

AC16 cardiomyocytes cultured on coverslips were fixed with 4% formaldehyde for 30 min at room temperature, permeabilized with 0.1% Triton X-100 in PBS for 10 min, and blocked with 5% BSA for 1.5 h at 37 °C. Cells were incubated overnight at 4 °C with rabbit anti-Nrf2 (1:50, 1639–1-AP, Proteintech, USA), followed by a 2 h incubation with goat anti-rabbit fluorescent secondary antibody at room temperature. Nuclei were counterstained with DAPI for 5 min. Fluorescence images were captured using a fluorescence microscope, and Nrf2 nuclear translocation was assessed by comparing nuclear and cytoplasmic fluorescence intensities.

### 2.8 Animal experiments

Male C57BL/6 mice (8–10 weeks old, 21–25 g) were obtained from an accredited Experimental Animal Center and maintained under specific pathogen-free conditions with controlled temperature (25 ± 2 °C), humidity (60 ± 10%), and a 12-h light/dark cycle. Cardiac-specific overexpression of miR-200a-3p was achieved via a single tail vein injection of adeno-associated virus serotype 9 (AAV9-miR-200a-3p, 1 × 10¹¹ viral genomes in 100 μL sterile PBS). AAV9 was selected due to its well-documented cardiac tropism following systemic administration, enabling efficient gene delivery to myocardial tissue (Inagaki et al., 2006; Zincarelli et al., 2008). A negative control vector (AAV9-NC) was used in parallel. Three to four weeks after AAV administration, myocardial I/R injury was induced. Mice were anesthetized with isoflurane, intubated, and mechanically ventilated, with body temperature maintained at 37 °C. The left anterior descending coronary artery was occluded with a 7–0 silk suture for 30 min, followed by reperfusion for 24 h. Sham-operated mice underwent identical procedures without coronary ligation. Mice were randomly assigned to four groups (n = 8 per group): Sham, I/R, I/R + AAV-NC, and I/R + AAV-miR-200a-3p. At the end of reperfusion, mice were euthanized under deep anesthesia, and blood was collected for serum assays. Myocardial tissues were rapidly excised; portions were snap-frozen in liquid nitrogen for RNA and protein extraction, while others were fixed in 4% paraformaldehyde for histological and immunohistochemistry (IHC) analysis. All procedures were approved by the Institutional Animal Care and Use Committee of Sir Run Run Shaw Hospital, Zhejiang University School of Medicine (Approval No. SRRSH20250725031, Zhejiang, China) and conducted in accordance with the National Institutes of Health Guide for the Care and Use of Laboratory Animals.

### 2.9 Serum cardiac injury analysis

Serum samples were collected 24 h after reperfusion following blood coagulation at room temperature and centrifugation at 3,000 × g for 10 min. Cardiac injury was assessed by determining serum levels of cardiac troponin I (cTn-I) and creatine kinase-MB (CK-MB) using commercially available assay kits (cTn-I, Cat. No. H149; CK-MB, Cat. No. H197-1; Nanjing Jiancheng Bioengineering Institute, Nanjing, China) in accordance with the manufacturer’s protocols.

### 2.10 Oxidative stress assessment

Oxidative stress in cultured AC16 cardiomyocytes and mouse myocardial tissues was assessed by measuring malondialdehyde (MDA) content and superoxide dismutase (SOD) activity. Cells were washed with ice-cold PBS, lysed, and centrifuged at 12,000 × g for 10 min at 4 °C, while myocardial tissues collected 24 h after reperfusion were homogenized in ice-cold saline (1:9, w/v) and centrifuged to obtain supernatants. MDA and SOD were measured using thiobarbituric acid–based (A003-1) and WST-1–based (A001-3) assay kits (Nanjing Jiancheng Bioengineering Institute), with results expressed as nmol/mg protein and U/mg protein.

### 2.11 ELISA assay

Levels of TNF-α and IL-1β in AC16 cell culture supernatants and mouse serum were measured by ELISA. Cell culture media were collected and centrifuged at 1,000 × g for 10 min at 4 °C, and mouse blood samples were collected 24 h after reperfusion, allowed to clot, and centrifuged at 3,000 × g for 10 min to obtain serum. Cytokines were quantified using commercially available ELISA kits (human TNF-α: DTA00D; human IL-1β: DLB50; mouse TNF-α: MTA00B; mouse IL-1β: MLB00C; R&D Systems) according to the manufacturers’ instructions.

### 2.12 H&E staining

Myocardial tissues excised were fixed in 4% paraformaldehyde for 24 h, dehydrated through graded ethanol, cleared in xylene, and embedded in paraffin. Sections (5 μm) were cut, mounted on slides, deparaffinized, and rehydrated. Hematoxylin and eosin (H&E) staining was performed using a commercial kit (Beyotime, H&E Staining Kit, C0105) following the manufacturer’s instructions. Briefly, slides were stained with hematoxylin, differentiated in 1% acid alcohol, blued in 0.2% ammonia water, and counterstained with eosin. After dehydration and mounting, images were captured under a light microscope (Olympus BX53). Cardiomyocyte arrangement, interstitial edema, and inflammatory cell infiltration were evaluated in a blinded manner, with five randomly selected fields per section quantified using ImageJ software.

### 2.13 TUNEL staining

Paraffin-embedded myocardial sections obtained as above were deparaffinized, rehydrated, and treated with proteinase K (20 μg/mL; Sigma−Aldrich, P6556) for 20 min at room temperature to facilitate antigen retrieval. Apoptotic cells were detected using a TUNEL assay kit (Roche, Cat. No. 11684817910) according to the manufacturer’s instructions, and nuclei were counterstained with DAPI (Sigma−Aldrich, D9542). Fluorescent images were captured under a fluorescence microscope (Zeiss Axio Observer). The extent of cardiomyocyte apoptosis was quantified in a blinded manner by calculating the percentage of TUNEL-positive nuclei relative to the total number of nuclei in each field.

### 2.14 Immunohistochemistry analysis

Myocardial tissues prepared as described above were deparaffinized, rehydrated, and subjected to antigen retrieval in citrate buffer (10 mM, pH = 6.0) using a microwave for 10 min. Endogenous peroxidase activity was blocked with 3% hydrogen peroxide for 10 min, and non-specific binding was blocked with 5% BSA (Sigma−Aldrich, A7906) for 30 min. Sections were incubated overnight at 4 °C with anti-KEAP1 primary antibody (Cell Signaling Technology, Cat. No. 8047, 1:200), followed by HRP-conjugated goat anti-rabbit secondary antibody (Abcam, ab6721, 1:500) for 1 h at room temperature. KEAP1 immunoreactivity was visualized with DAB substrate (Vector Laboratories, SK-4100) and counterstained with hematoxylin. KEAP1 immunoreactivity was quantified as mean optical density (MOD, IOD/area) using ImageJ. Five randomly selected non-overlapping fields per section were analyzed by observers blinded to group allocation.

### 2.15 Quantitative real-time PCR

Total RNA was isolated from cells or myocardial tissues using TRIzol reagent (Invitrogen, Carlsbad, CA, USA) following the manufacturer’s instructions. For miR-200a-3p detection, RNA was reverse-transcribed using the miRcute Plus miRNA First-Strand cDNA Kit and amplified with the miRcute Plus miRNA First-Strand cDNA Kit and amplified with the miRcute Plus miRNA qPCR Kit (SYBR Green, Tiangen Biotech, Beijing, China), with U6 serving as an internal control. For KEAP1 mRNA analysis, complementary DNA (cDNA) was synthesized using the PrimeScript RT Reagent Kit (Takara, Shiga, Japan), and quantitative real-time PCR was performed with SYBR Green Master Mix (Takara), using GAPDH as a reference gene. Relative expression levels were calculated using the 2^-ΔΔCt^ method. The primer sequences are shown in **Table 1**. All reactions were run in triplicate, and experiments were repeated at least three times independently.

**Table 1:** Primers for quantitative real-time PCR.

Gene	Forward (5′ - 3′)	Reverse (5′ - 3′)
miR-200a-3p	CTTACCGGACAGTGCTG	GAACATGTCTGCGTATCTC
U6	CTCGCTTCGGCAGCACAT	TTTGCGTGTCATCCTTGCG
KEAP1	ACGCGCAGCGATGGAG	TCCTCCAGGGTGTAGCTGAA
GAPDH	GTCTCCTCTGACTTCAACAGCG	ACCACCCTGTTGCTGTAGCCAA

### 2.16 Western blot analysis

Total protein was extracted from AC16 cells or mouse myocardial tissues using RIPA lysis buffer (Beyotime) supplemented with protease and phosphatase inhibitors. For nuclear and cytoplasmic protein separation, the commercial Nuclear and Cytoplasmic Extraction Kit (Thermo Fisher Scientific) was used according to the manufacturer’s instructions. Protein concentrations were measured using a BCA assay kit (Beyotime). Equal amounts of protein (30 μg per lane) were resolved by 10–12% SDS-PAGE and transferred onto PVDF membranes (Millipore). Membranes were blocked with 5% non-fat milk in TBST (Tris-buffered saline with 0.1% Tween-20) for 1 h at room temperature, followed by overnight incubation at 4 °C with primary antibodies against KEAP1 (1:1000, #8047, Cell signaling), Nrf2 (1:1000, #12721, Cell signaling), HO-1 (1:1000, #70081, Cell signaling), NQO1 (1:1000, #62262, Cell signaling), NLRP3 (1:1000, ab263899, Abcam), cleaved caspase-3 (1:1000, #9664, Cell signaling), and GAPDH (1:5000, #5174, Cell signaling) or Lamin B1 (1:1000, #13435, Cell signaling) for nuclear fractions at recommended dilutions. After washing, membranes were incubated with HRP-conjugated secondary antibodies for 1 h at room temperature. Protein signals were detected using enhanced chemiluminescence (ECL; Thermo Fisher Scientific) and quantified with ImageJ software. For Nrf2, the nuclear-to-cytoplasmic ratio was calculated by normalizing nuclear Nrf2 to Lamin B1 and cytoplasmic Nrf2 to GAPDH. All experiments were performed in triplicate.

### 2.17 Statistical analysis

All data are presented as mean ± standard deviation (SD), derived from at least three independent *in vitro* experiments or from all animals within each *in vivo* group. Statistical comparisons between two groups were performed using an unpaired Student’s t-test. For analyses involving more than two groups, one-way ANOVA followed by Tukey’s *post hoc* test was applied. A *p*-value of less than 0.05 was considered statistically significant. Analyses were conducted using GraphPad Prism 8.0 (GraphPad Software, San Diego, CA, USA).

## 3 Results

### 3.1 MiR-200a-3p mitigated H/R-induced oxidative injury in AC16 cardiomyocytes

**Figure 1:**
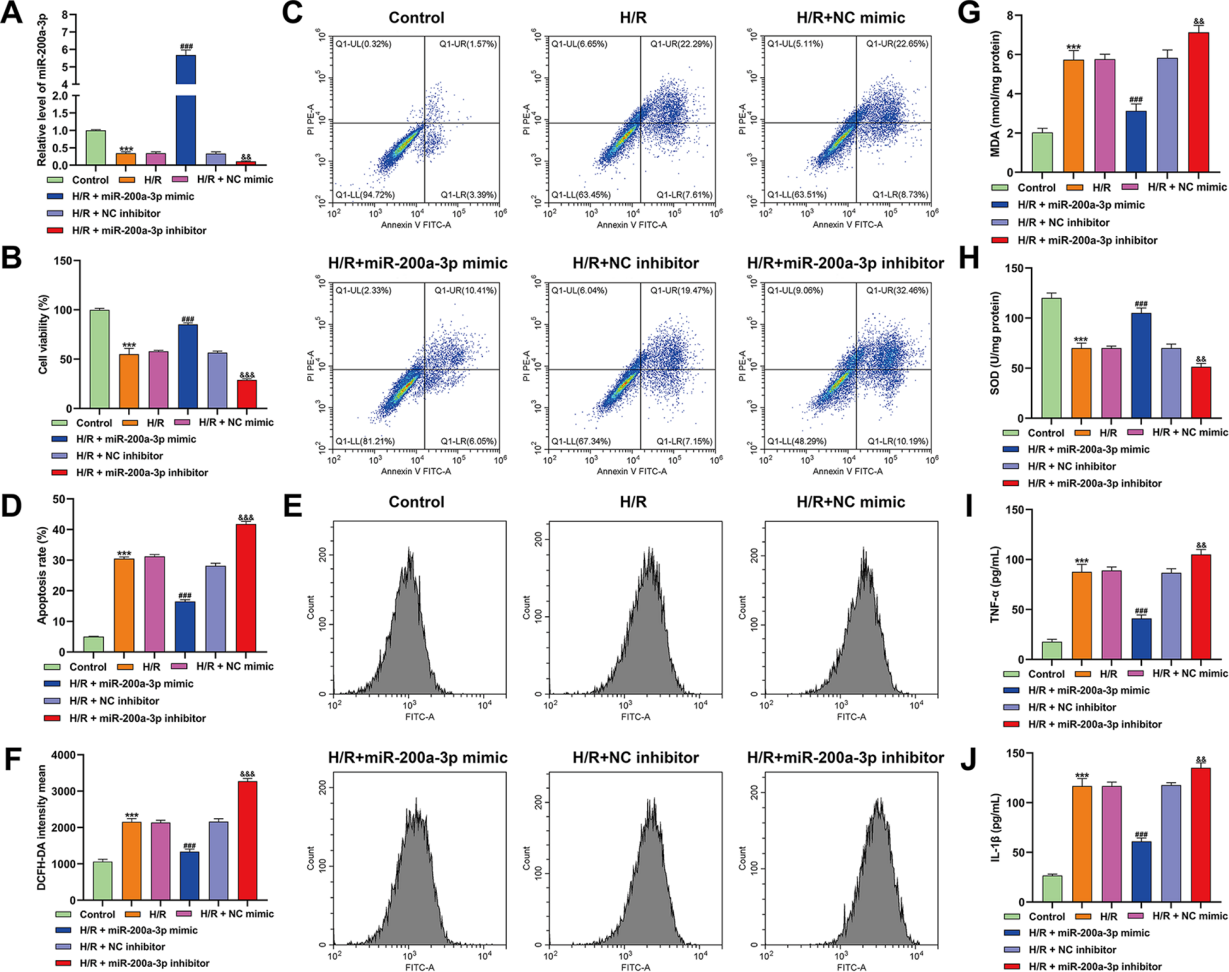
Assessment of miR-200a-3p function in AC16 cardiomyocytes under hypoxia–reoxygenation (H/R). **(A)** miR-200a-3p expression was quantified by quantitative real-time PCR following transfection with miR-200a-3p mimic, inhibitor, or corresponding negative controls under normoxic or H/R conditions. **(B)** Cell viability was measured using the CCK-8 assay. **(C, D)** Apoptosis was analyzed by flow cytometry using Annexin V-FITC/PI staining. **(E, F)** Intracellular reactive oxygen species (ROS) levels were assessed using the DCFH-DA fluorescent probe with flow cytometric analysis. **(G, H)** Oxidative stress markers, including malondialdehyde (MDA) content and superoxide dismutase (SOD) activity, were determined in cell lysates. **(I, J)** Secretion of inflammatory cytokines TNF-α and IL-1β into culture supernatants was measured by ELISA assay. Data are presented as mean ± standard deviation (SD) from at least three independent experiments. Statistical comparisons were performed using one-way ANOVA followed by Tukey’s *post hoc* test. ***p < 0.001, *vs.* Control, ^###^*p* < 0.001, *vs.* H/R + NC mimic, &&*p* < 0.01, ^&&&^*p* < 0.001, *vs.* H/R + NC inhibitor.

To explore the protective role of miR-200a-3p in cardiomyocyte injury, its expression was first examined under H/R conditions. As shown in **Figure 1A**, H/R significantly decreased miR-200a-3p levels, suggesting that downregulation of this miRNA may contribute to cellular damage. Transfection with a miR-200a-3p mimic effectively restored its expression, whereas inhibition further reduced its levels, confirming successful modulation. Functional analyses revealed that H/R markedly reduced cell viability (**Figure 1B**) and increased apoptosis (**Figures 1C, D**). Overexpression of miR-200a-3p attenuated these detrimental effects, while its inhibition further exacerbated cell death, suggesting a cytoprotective role. H/R also induced excessive ROS production (**Figures 1E, F**) and disrupted oxidative balance, as reflected by elevated MDA levels and decreased SOD activity (**Figures 1G, H**). These oxidative changes were largely reversed by miR-200a-3p mimic and worsened by inhibitor treatment. In parallel, inflammatory responses were assessed by measuring TNF-α and IL-1β in culture supernatants. H/R markedly increased both cytokines (**Figures 1I, J**), while miR-200a-3p overexpression significantly reduced their levels, and inhibition amplified the inflammatory response. Collectively, these findings demonstrate that miR-200a-3p protects AC16 cardiomyocytes against H/R-induced injury by enhancing cell survival, reducing apoptosis and oxidative stress, and attenuating inflammatory responses.

### 3.2 KEAP1 was identified as a target of miR-200a-3p

**Figure 2:**
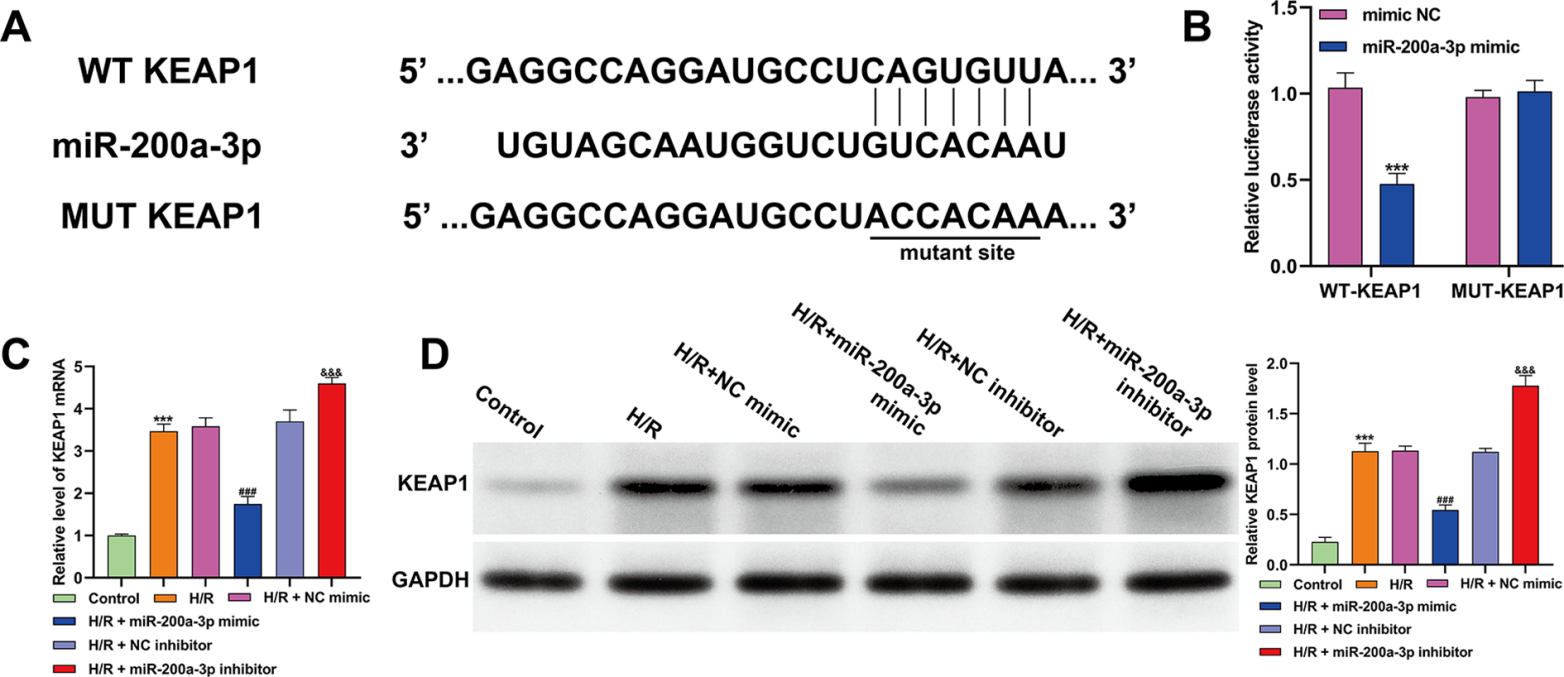
Validation of KEAP1 as a direct target of miR-200a-3p. **(A)** Schematic representation of the predicted binding site of miR-200a-3p within the 3′-UTR of human KEAP1 mRNA, as identified by TargetScan. Both the wild-type (WT) and mutant (MUT) sequences used for dual-luciferase reporter assays are shown. **(B)** Dual-luciferase reporter assays were performed in HEK293T cells co-transfected with WT- or MUT-KEAP1 3′-UTR plasmids and either miR-200a-3p mimic or NC mimic. Firefly luciferase activity was normalized to Renilla luciferase activity, and relative luciferase activity was calculated. Statistical analyses were performed using Student’s t-test. ***p < 0.001, *vs.* NC mimic. **(C, D)** AC16 cells were transfected with miR-200a-3p mimic, inhibitor, or their corresponding controls and then subjected to H/R treatment. KEAP1 expression was assessed by quantitative real-time PCR **(C)** and western blotting **(D)**. Protein bands were quantified using ImageJ software, and GAPDH was used as the internal reference. All data are presented as mean ± SD from at least three independent experiments. Statistical analyses were performed using one-way ANOVA with Tukey’s *post hoc* test. ***p < 0.001, *vs.* control; ^###^*p* < 0.001, *vs.* H/R + NC mimic, ^&&^*p* < 0.01, ^&&&^*p* < 0.001, *vs.* H/R + NC inhibitor.

KEAP1 is a key regulator of apoptosis, oxidative stress, and inflammatory responses. Bioinformatic prediction using TargetScan (http://www.targetscan.org/vert_71/) identified a conserved binding site for miR-200a-3p within the 3′-UTR of KEAP1 (**Figure 2A**). To experimentally validate this interaction, dual-luciferase reporter assays were performed in HEK293T cells co-transfected with either WT or MUT KEAP1 3′-UTR constructs and miR-200a-3p mimic or negative control. Overexpression of miR-200a-3p significantly reduced the relative luciferase activity of the WT-KEAP1 reporter, whereas no significant change was observed for the MUT reporter (**Figure 2B**), supporting a direct interaction between miR-200a-3p and KEAP1 3′-UTR. To examine whether miR-200a-3p regulates KEAP1 expression under H/R conditions, AC16 cardiomyocytes were transfected with miR-200a-3p mimic, inhibitor, or corresponding controls and subjected to H/R injury. Quantitative PCR and western blot analyses showed that miR-200a-3p overexpression significantly decreased KEAP1 mRNA and protein levels, while miR-200a-3p inhibition increased KEAP1 expression compared with H/R + NC groups (**Figures 2C, D**). Collectively, these findings indicate that miR-200a-3p was found to be target KEAP1 and negatively regulates its expression in cardiomyocytes under H/R stress.

### 3.3 KEAP1 contributed to the protective effects of miR-200a-3p against H/R-induced oxidative injury in cardiomyocytes

**Figure 3:**
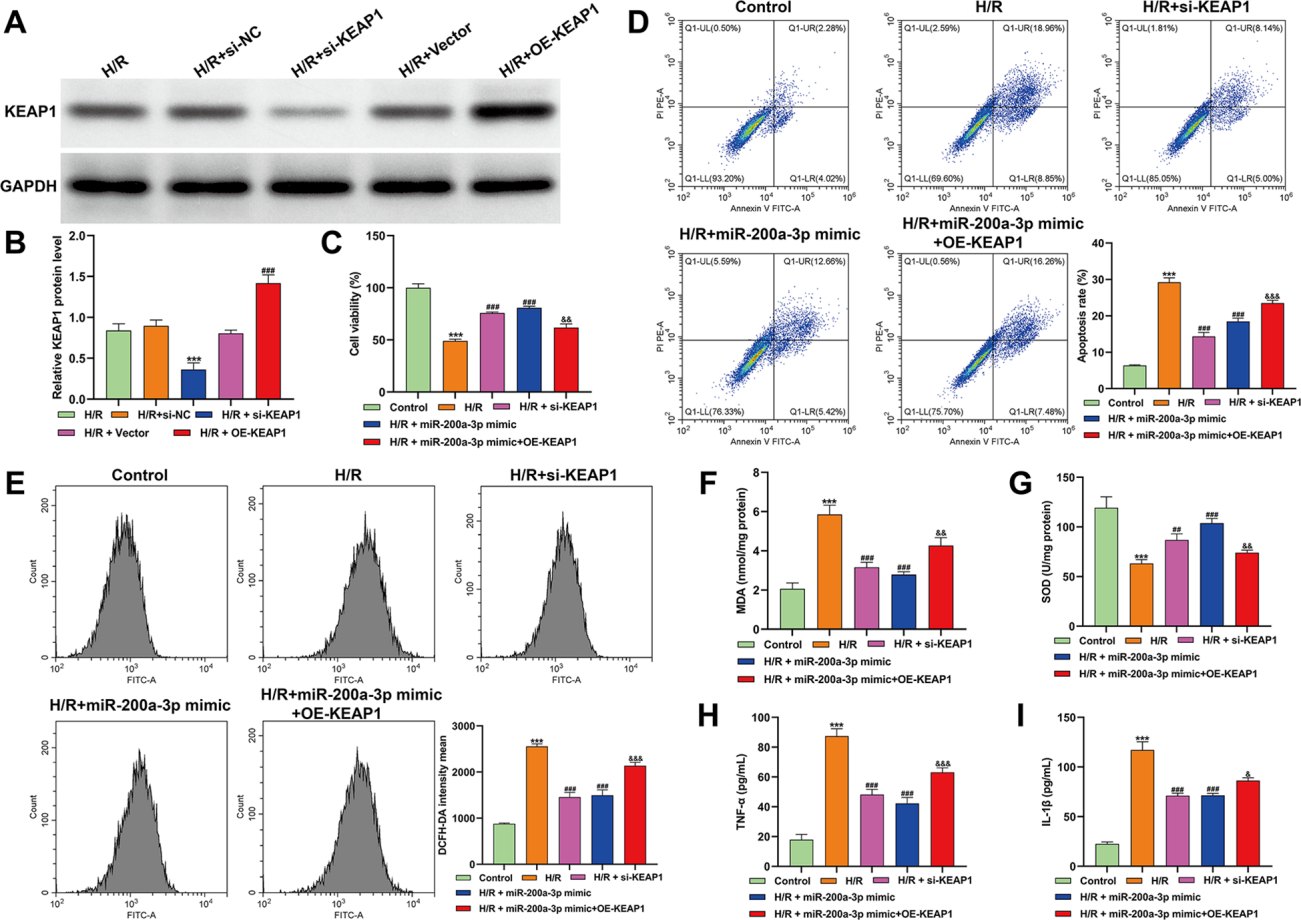
KEAP1 mediates the protective effects of miR-200a-3p against H/R-induced oxidative injury in AC16 cardiomyocytes. **(A, B)** Western blot analysis of KEAP1 protein expression in AC16 cardiomyocytes transfected with si-KEAP1 or KEAP1 overexpression plasmid under H/R conditions, confirming the efficiency of KEAP1 knockdown and overexpression. ***p < 0.001, *vs.* H/R + si-NC; ^###^*p* < 0.001, *vs.* H/R + Vector; **(C)** Cell viability assessed by CCK-8 assay in AC16 cardiomyocytes. **(D)** Flow cytometric analysis of apoptosis using Annexin V/PI staining in AC16 cardiomyocyte. **(E)** Intracellular ROS levels measured by flow cytometry in AC16 cardiomyocytes. **(F, G)** Oxidative stress–related biochemical indices, including MDA levels **(F)** and SOD activity **(G)**, in AC16 cardiomyocytes under the indicated experimental conditions. **(H, I)** ELISA analysis of pro-inflammatory cytokines TNF-α **(H)** and IL-1β **(I)** in culture supernatants of AC16 cardiomyocyte. Data are presented as mean ± SD from at least three independent experiments. Statistical analyses were performed using one-way ANOVA with Tukey’s *post hoc* test. ***p < 0.001, *vs.* control; ^###^*p* < 0.001, *vs.* H/R, ^&^*p* < 0.05, ^&&^*p* < 0.01, ^&&&^*p* < 0.001, *vs.* H/R + miR-200a-3p mimic.

To clarify whether KEAP1 functionally mediates the cytoprotective effects of miR-200a-3p under H/R stress, rescue experiments were conducted in AC16 cardiomyocytes. Western blot analysis confirmed efficient modulation of KEAP1 expression, with siRNA markedly reducing and plasmid-mediated overexpression significantly increasing KEAP1 protein levels in H/R-treated cells (**Figures 3A, B**). Functional assays revealed that H/R exposure substantially impaired cardiomyocyte viability, whereas either KEAP1 silencing or miR-200a-3p overexpression significantly restored cell survival. Importantly, enforced KEAP1 overexpression largely abolished the pro-survival effect of the miR-200a-3p mimic, resulting in viability levels comparable to those observed under H/R alone (**Figure 3C**). Consistently, flow cytometric analysis demonstrated that H/R markedly increased apoptotic cell death. This effect was significantly attenuated by KEAP1 knockdown or miR-200a-3p overexpression, while reintroduction of KEAP1 reversed the anti-apoptotic action of miR-200a-3p (**Figure 3D**). Given the central role of oxidative stress in H/R injury, intracellular redox status was further evaluated. H/R-induced ROS overproduction was significantly suppressed by KEAP1 silencing or miR-200a-3p mimic treatment, whereas KEAP1 overexpression largely restored ROS accumulation despite miR-200a-3p upregulation (**Figure 3E**). In line with these findings, alterations in lipid peroxidation and antioxidant capacity—reflected by elevated MDA levels and reduced SOD activity—were effectively normalized by KEAP1 knockdown or miR-200a-3p overexpression but were significantly reversed upon KEAP1 overexpression (**Figures 3F, G**). Inflammatory responses were assessed in parallel. ELISA results showed that H/R markedly increased TNF-α and IL-1β secretion, which was significantly reduced by KEAP1 silencing or miR-200a-3p overexpression. Notably, KEAP1 overexpression largely negated the anti-inflammatory effects conferred by miR-200a-3p (**Figures 3H, I**). Collectively, these rescue experiments provide compelling functional evidence that KEAP1 is a critical downstream mediator of miR-200a-3p, governing its protective effects against H/R-induced oxidative injury in cardiomyocytes.

### 3.4 MiR-200a-3p promoted Nrf2/ARE signaling by targeting KEAP1 in cardiomyocytes

**Figure 4:**
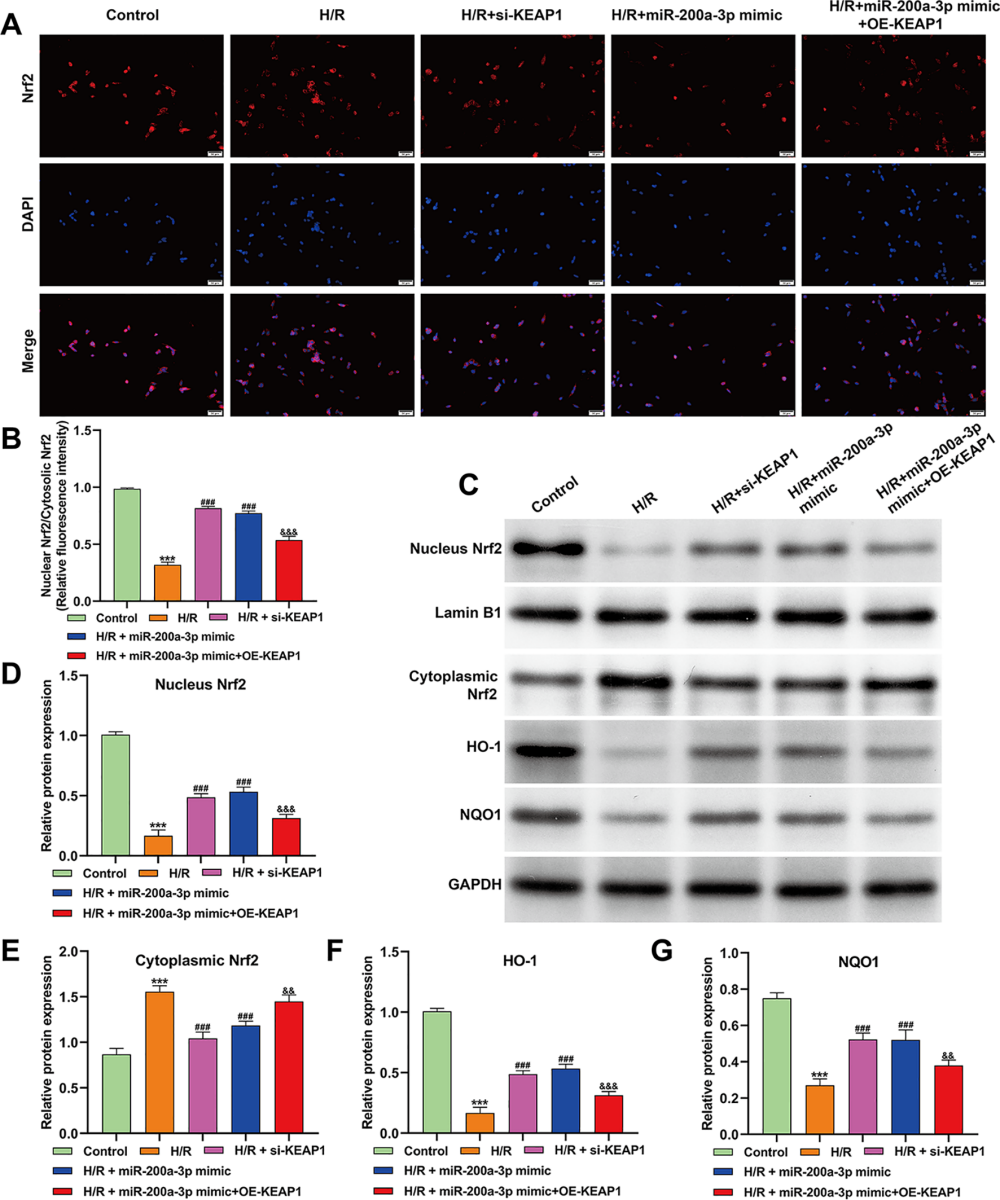
MiR-200a-3p promotes Nrf2/ARE signaling by targeting KEAP1 in cardiomyocytes. **(A, B)** Immunofluorescence staining of Nrf2 in AC16 cardiomyocytes under the indicated conditions. Representative images show Nrf2 localization in the nucleus and cytoplasm following H/R injury with miR-200a-3p overexpression, KEAP1 silencing, or combined miR-200a-3p mimic and KEAP1 overexpression. Nuclei were counterstained with DAPI. **(C–G)** Western blot analysis of nuclear and cytoplasmic Nrf2, as well as the Nrf2 downstream antioxidant enzymes HO-1 and NQO1, in AC16 cardiomyocytes subjected to H/R and genetic modulation of miR-200a-3p and KEAP1. Data are presented as mean ± SD from at least three independent experiments. Statistical analyses were performed using one-way ANOVA with Tukey’s *post hoc* test. ***p < 0.001, *vs.* control; ^###^*p* < 0.001, *vs.* H/R, ^&^*p* < 0.05, ^&&^*p* < 0.01, ^&&&^*p* < 0.001, *vs.* H/R + miR-200a-3p mimic.

To further define the downstream mechanism of the miR-200a-3p/KEAP1 axis, Nrf2/ARE pathway activation was examined in AC16 cardiomyocytes following H/R injury. Immunofluorescence analysis showed that H/R markedly impaired Nrf2 nuclear localization, whereas miR-200a-3p overexpression significantly enhanced Nrf2 nuclear translocation. This effect was largely reversed by KEAP1 overexpression, while KEAP1 silencing phenocopied the action of the miR-200a-3p mimic (**Figures 4A, B**). Consistently, western blot analysis revealed that H/R reduced nuclear Nrf2 levels and increased cytoplasmic Nrf2 expression. Both miR-200a-3p overexpression and KEAP1 knockdown effectively restored Nrf2 nuclear accumulation, whereas KEAP1 overexpression markedly attenuated this effect (**Figures 4C–E**). In parallel, the expression of Nrf2 downstream antioxidant enzymes was assessed. H/R significantly suppressed HO-1 and NQO1 protein levels, which were restored by miR-200a-3p mimic or KEAP1 silencing but abolished by KEAP1 overexpression (**Figures 4C, F, G**). Together, these results demonstrate that miR-200a-3p activates the Nrf2/ARE antioxidant pathway by suppressing KEAP1, thereby enhancing antioxidant defense in cardiomyocytes under H/R stress.

### 3.5 MiR-200a-3p overexpression attenuated myocardial I/R injury *in vivo*

**Figure 5:**
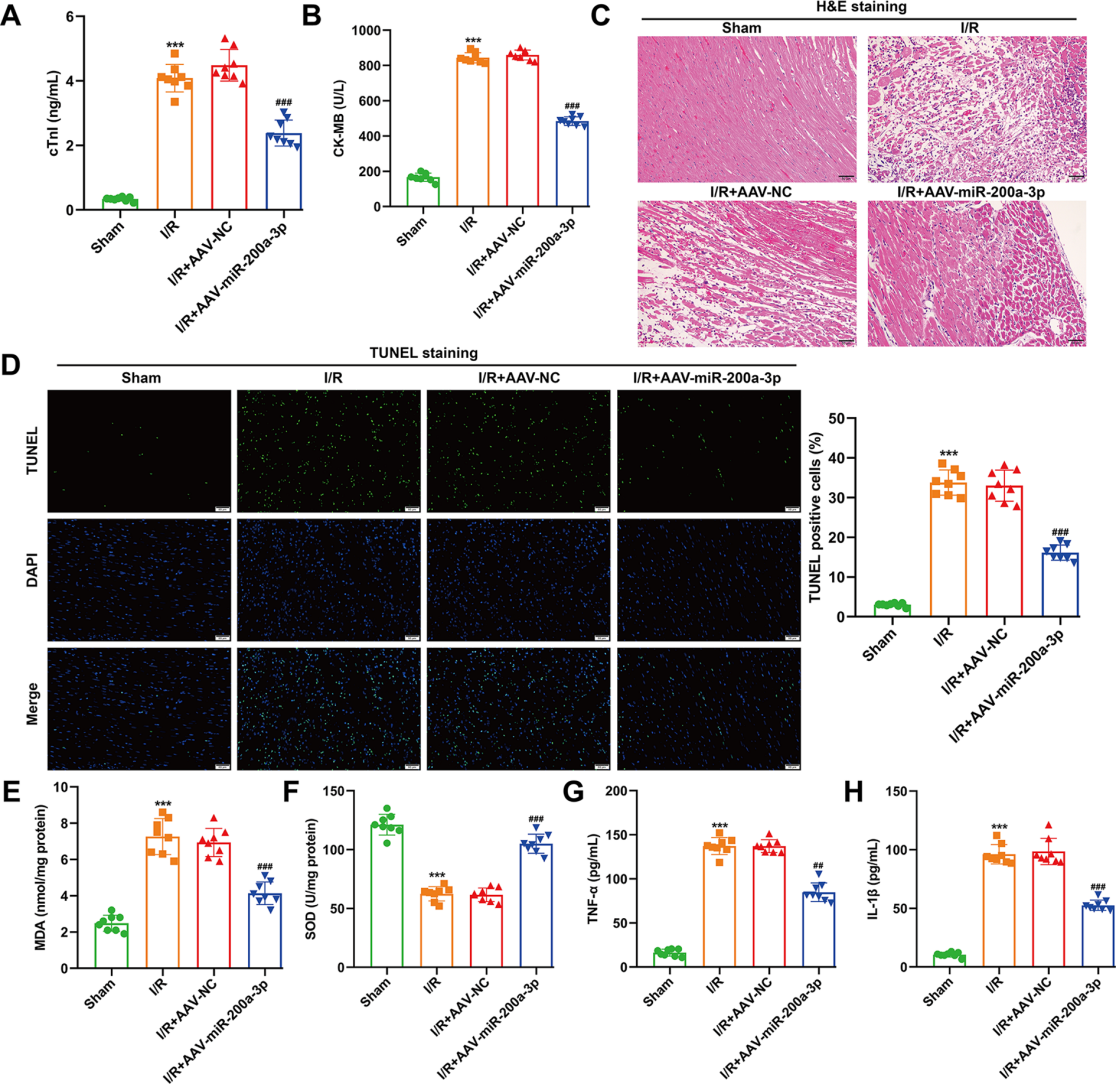
Cardiac overexpression of miR-200a-3p attenuates myocardial ischemia–reperfusion (I/R) injury in mice. **(A, B)** Serum levels of cardiac injury markers cTnI **(A)** and CK-MB **(B)** in sham-operated mice, I/R mice, I/R + AAV-NC, and I/R + AAV-miR-200a-3p groups. **(C)** Representative H&E staining of myocardial sections showing tissue architecture, including cardiomyocyte arrangement, interstitial edema, and inflammatory cell infiltration. Scale bar = 50 μm; **(D)** TUNEL staining of myocardial sections to assess cardiomyocyte apoptosis under the indicated treatments. Scale bar = 50 μm; **(E, F)** Myocardial oxidative stress markers, including MDA **(E)** and SOD activity **(F)**, in the different experimental groups. **(G, H)** Serum levels of pro-inflammatory cytokines TNF-α **(G)** and IL-1β **(H)** measured by ELISA. Data are presented as mean ± SD (n = 8 per group). ***p < 0.001, *vs.* sham; ^##^*p* < 0.01, ^###^*p* < 0.001, *vs.* I/R + AAV-NC.

To validate the cardioprotective role of miR-200a-3p *in vivo*, a murine myocardial I/R model was established with cardiac-targeted AAV9-mediated miR-200a-3p overexpression. Serum markers of myocardial injury were markedly increased after I/R, as evidenced by elevated cTnI and CK-MB levels, which were significantly reduced by miR-200a-3p overexpression compared with I/R and AAV-NC–treated mice (**Figures 5A, B**). Histological analysis via H&E staining revealed that I/R caused severe myocardial structural disruption, including myofibrillar disorganization, interstitial edema, and inflammatory infiltration, whereas these pathological changes were substantially alleviated in the miR-200a-3p overexpression group (**Figure 5C**). Consistently, TUNEL staining demonstrated a significant increase in cardiomyocyte apoptosis following I/R, which was markedly reduced by miR-200a-3p overexpression (**Figure 5D**). Assessment of myocardial oxidative stress showed that I/R significantly increased MDA levels and decreased SOD activity. These alterations were effectively reversed by miR-200a-3p overexpression, indicating restoration of antioxidant capacity in the ischemic myocardium (**Figures 5E, F**). In parallel, inflammatory responses were evaluated. I/R markedly elevated serum TNF-α and IL-1β levels, both of which were significantly suppressed by miR-200a-3p overexpression (**Figures 5G, H**). Together, these findings demonstrate that cardiac overexpression of miR-200a-3p mitigates myocardial injury, apoptosis, oxidative stress, and inflammation following I/R *in vivo*, and the consistent improvement across multiple injury-related parameters supports a functionally relevant cardioprotective effect *in vivo*.

### 3.6 MiR-200a-3p overexpression activated the KEAP1–Nrf2 antioxidant pathway to mediate cardioprotection *in vivo*

**Figure 6:**
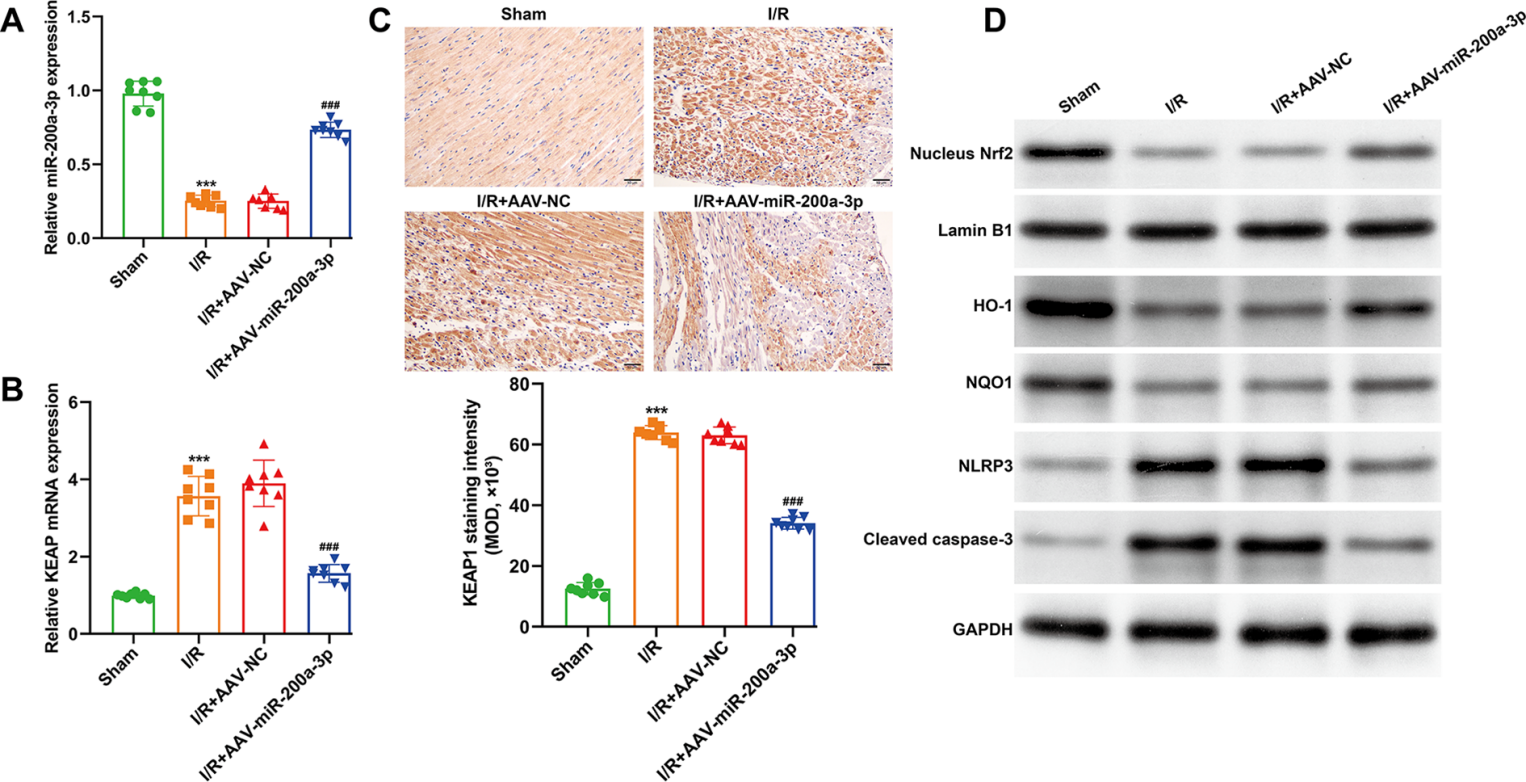
MiR-200a-3p activates the KEAP1–Nrf2 antioxidant pathway and attenuates inflammation in mice after I/R. **(A, B)** Quantitative real-time PCR analysis of miR-200a-3p **(A)** and KEAP1 mRNA **(B)** levels in myocardial tissue from sham, I/R, I/R + AAV-NC, and I/R + AAV-miR-200a-3p groups. **(C)** Immunohistochemical staining of KEAP1 in myocardial sections after I/R and reduction following miR-200a-3p overexpression and quantitative analysis of KEAP1 expression expressed as mean optical density (MOD, ×10³). Scale bar = 50 μm; **(D)** Western blot analysis of nuclear Nrf2, HO-1, NQO1, and inflammation/apoptosis-related markers NLRP3 and cleaved caspase-3 in myocardial tissue from the indicated groups. Data are presented as mean ± SD (n = 8 per group). ***p < 0.001, *vs.* sham; ^##^*p* < 0.01, ^###^*p* < 0.001, *vs.* I/R + AAV-NC.

To investigate whether miR-200a-3p confers cardioprotection via the KEAP1–Nrf2 axis *in vivo*, miR-200a-3p expression and KEAP1–Nrf2 pathway activation were assessed in myocardial tissue following I/R. Quantitative real-time PCR confirmed effective cardiac overexpression of miR-200a-3p in the AAV9-miR-200a-3p group (**Figure 6A**), which was associated with a significant reduction in KEAP1 mRNA compared with I/R and I/R + AAV-NC groups (**Figure 6B**). IHC analysis further demonstrated increased KEAP1 protein expression in myocardial tissue following I/R, which was markedly attenuated by miR-200a-3p overexpression (**Figure 6C**). Western blot analysis revealed that I/R significantly decreased nuclear Nrf2 levels and downregulated its downstream antioxidant targets HO-1 and NQO1. Overexpression of miR-200a-3p restored Nrf2 nuclear accumulation and enhanced HO-1 and NQO1 expression, indicating activation of the antioxidant response (**Figure 6D**). In parallel, myocardial inflammation and apoptosis were evaluated. I/R significantly increased the expression of NLRP3 and cleaved caspase-3, whereas miR-200a-3p overexpression markedly suppressed these inflammatory and apoptotic markers (**Figure 6D**), consistent with improved cardiac protection. These findings indicate that miR-200a-3p-mediated myocardial protection is closely linked to KEAP1 inhibition, activation of Nrf2 signaling, and modulation of oxidative stress and inflammatory responses.

## 4 Discussion

Myocardial I/R injury remains a major clinical challenge due to its high incidence and the lack of targeted therapies that effectively reduce post−ischemic cardiac damage (Xiang et al., 2024). It is characterized by excessive oxidative stress, inflammatory activation, and cardiomyocyte apoptosis, which collectively drive functional deterioration and adverse remodeling (Luo et al., 2025). Among these, oxidative stress is a central driver, and the KEAP1–NRF2 axis is a key endogenous defense mechanism maintaining redox homeostasis in cardiomyocytes (Shen et al., 2019). However, upstream regulators of KEAP1–NRF2 during I/R injury remain incompletely understood. In this study, we show that miR-200a-3p is downregulated in H/R-treated cardiomyocytes and ischemic myocardium, with expression inversely correlated with oxidative and inflammatory markers, consistent with stress-induced miRNA dysregulation in cardiac injury models (Sun and Zhang, 2019).

Previous studies indicate that miR-200a-3p modulates oxidative stress and inflammation in diverse contexts. For example, it reduces inflammatory signaling and oxidative damage in ulcerative colitis via KEAP1–NRF2 activation (Peng et al., 2023) and regulates redox balance in cancer and diabetic kidney disease models (Eades et al., 2011; Wei et al., 2014b). In cardiovascular contexts, it protects against diabetic cardiomyopathy (You et al., 2023) and modulates stress-induced oxidative and inflammatory responses in cardiomyocytes (Ma et al., 2021). Despite these observations, most prior studies have either focused on non-cardiac systems or described phenotypic effects without directly establishing a mechanistic link between miR-200a-3p and KEAP1–NRF2 signaling in cardiomyocytes during I/R injury. Our findings extend these observations by demonstrating, both *in vitro* and *in vivo*, that miR-200a-3p directly targets KEAP1, activates NRF2, and enhances downstream antioxidant defenses during myocardial I/R. Notably, miR-200a-3p can exhibit context-dependent effects, as it aggravates doxorubicin-induced cardiotoxicity via PEG3 and SIRT1/NF-κB signaling (Fu et al., 2021), suggesting that the biological effects of miR-200a-3p are context-dependent and influenced by the nature of cellular stress.

Mechanistically, KEAP1 sequestration normally limits NRF2 nuclear translocation and antioxidant gene transcription (Ulasov et al., 2022). Oxidative stress disrupts this complex, enabling NRF2-dependent induction of HO-1, NQO1, and SOD2, which collectively limit ROS accumulation (Mata and Cadenas, 2021; Li et al., 2022). Our findings suggest that miR-200a-3p may suppress KEAP1 in cardiomyocytes, thereby linking it to canonical NRF2-mediated antioxidant responses and indicating potential therapeutic relevance that warrants further investigation. MiR-200a-3p overexpression also reduced inflammatory cytokines, suggesting anti-inflammatory effects. While the exact mechanisms were not examined here, NRF2 is known to negatively regulate NF-κB–mediated inflammation in cardiac tissue (Wardyn et al., 2015; Kobayashi et al., 2016), indicating potential crosstalk that warrants future investigation. Furthermore, other cardioprotective miRNAs, such as miR-541-5p (Zhao et al., 2025) and miR-24 (Li et al., 2021), similarly mitigate oxidative stress and inflammation in I/R models, highlighting a shared mechanism of miRNA-mediated regulation of redox and inflammatory pathway.

Importantly, the identification of miR-200a-3p as a cardioprotective factor also suggests potential translational applications. Strategies such as viral vectors, lipid nanoparticles, or exosome-mediated delivery could enable cardiac-specific miRNA therapy, although off-target effects, immune responses, and long-term safety require careful evaluation in preclinical models (Moraes et al., 2021; Corradi et al., 2024). Despite these considerations, our *in vivo* data demonstrate that miR-200a-3p overexpression activates myocardial KEAP1–NRF2 signaling, augments antioxidant capacity, and suppresses inflammation and cell death, highlighting this axis as a central mechanism underlying its cardioprotective effects. These injury-related improvements are widely considered to reflect the severity of myocardial I/R damage and its functional consequences.

Despite these insights, several limitations should be acknowledged. First, although we demonstrate a functional role for miR−200a−3p in regulating the KEAP1–NRF2 pathway, the upstream mechanisms driving its downregulation during I/R remain unclear, and epigenetic or transcriptional factors may contribute. Second, while AC16 cells and C57BL/6 mice are well-established experimental models, AC16 cells may not fully reflect the metabolic and stress-response characteristics of primary cardiomyocytes, and species- or cell-type differences may affect translational relevance. Nonetheless, our *in vivo* I/R experiments support the physiological significance of the findings and mitigate some limitations of the *in vitro* system. Third, cardiac functional assessments—including echocardiography and infarct size quantification—were not performed due to experimental constraints. However, multiple established indicators of myocardial injury were consistently improved in this study, which are commonly associated with cardiac functional impairment in I/R models. These findings support a cardioprotective effect that is functionally relevant *in vivo*, although direct functional measurements were not performed. Future studies incorporating primary human cardiomyocytes, cardiac tissue models, and functional/longitudinal evaluations will be important to extend and validate these mechanistic insights.

**Figure 7:**
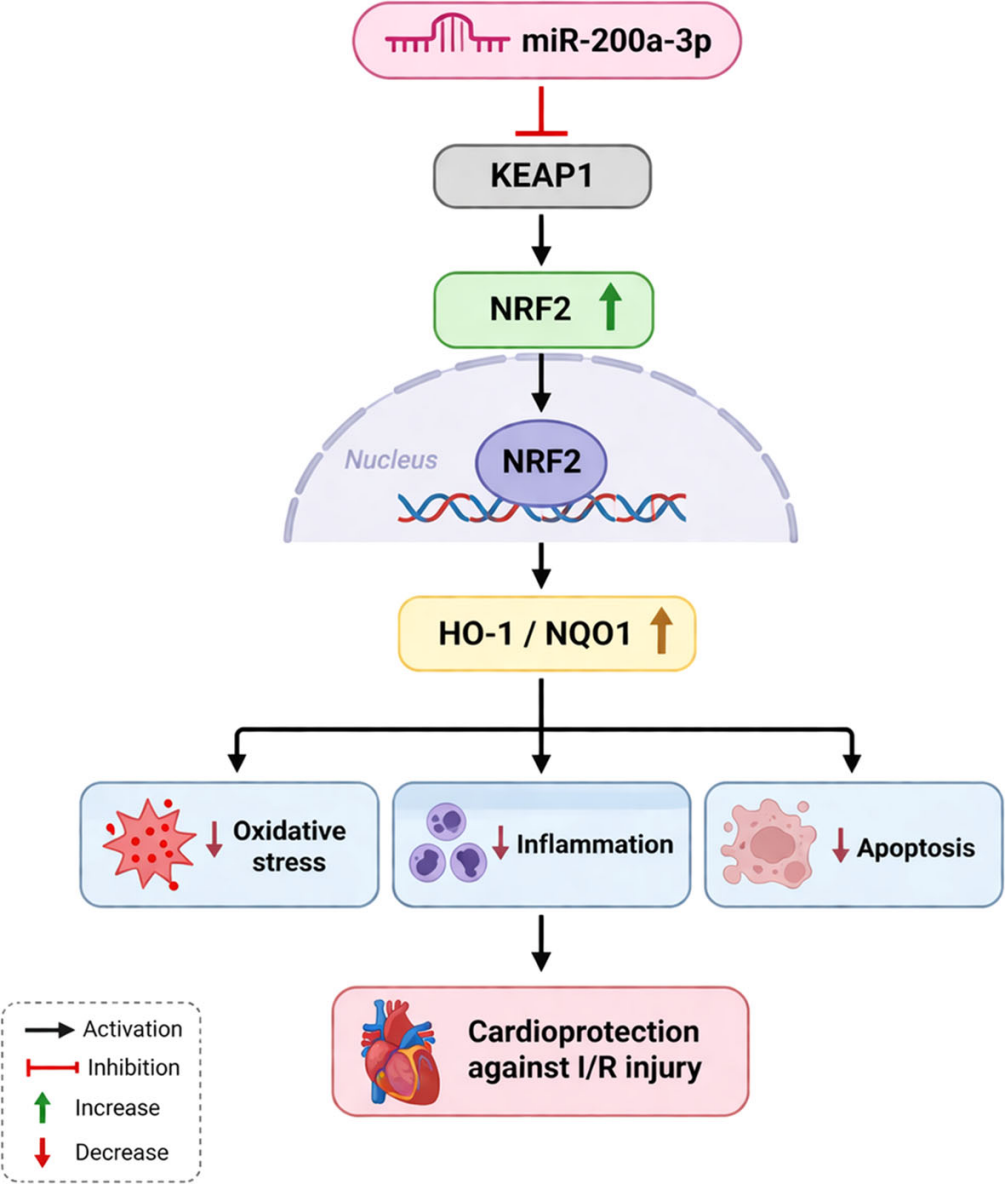
Graphical summary of action mechanism of miR-200a-3p/KEAP1-NRF2/ARE signaling against myocardial ischemia-reperfusion injury.

In conclusion, our results demonstrate that miR−200a−3p attenuates myocardial I/R injury by targeting KEAP1 to activate NRF2−dependent antioxidant defenses, simultaneously suppressing oxidative stress and inflammatory responses (**Figure 7**). These findings provide additional insight into the role of miR-200a-3p in myocardial I/R injury by implicating its involvement in the regulation of the KEAP1–NRF2 antioxidant pathway and its potential contribution to cardioprotection.

## Data Availability

The original contributions presented in the study are included in the article/**Supplementary Material**. Further inquiries can be directed to the corresponding authors.

## Supplementary material

Supplementary Figure S1 All the original and unprocessed protein bands.
