# Supplementary figures and images for: MiR-200a-3p protects against myocardial ischemia-reperfusion injury via KEAP1–NRF2 signaling
Source: Front Physiol. 2026 May 1;17:1826306. doi: 10.3389/fphys.2026.1826306 (PMC13175884; doi:10.3389/fphys.2026.1826306)

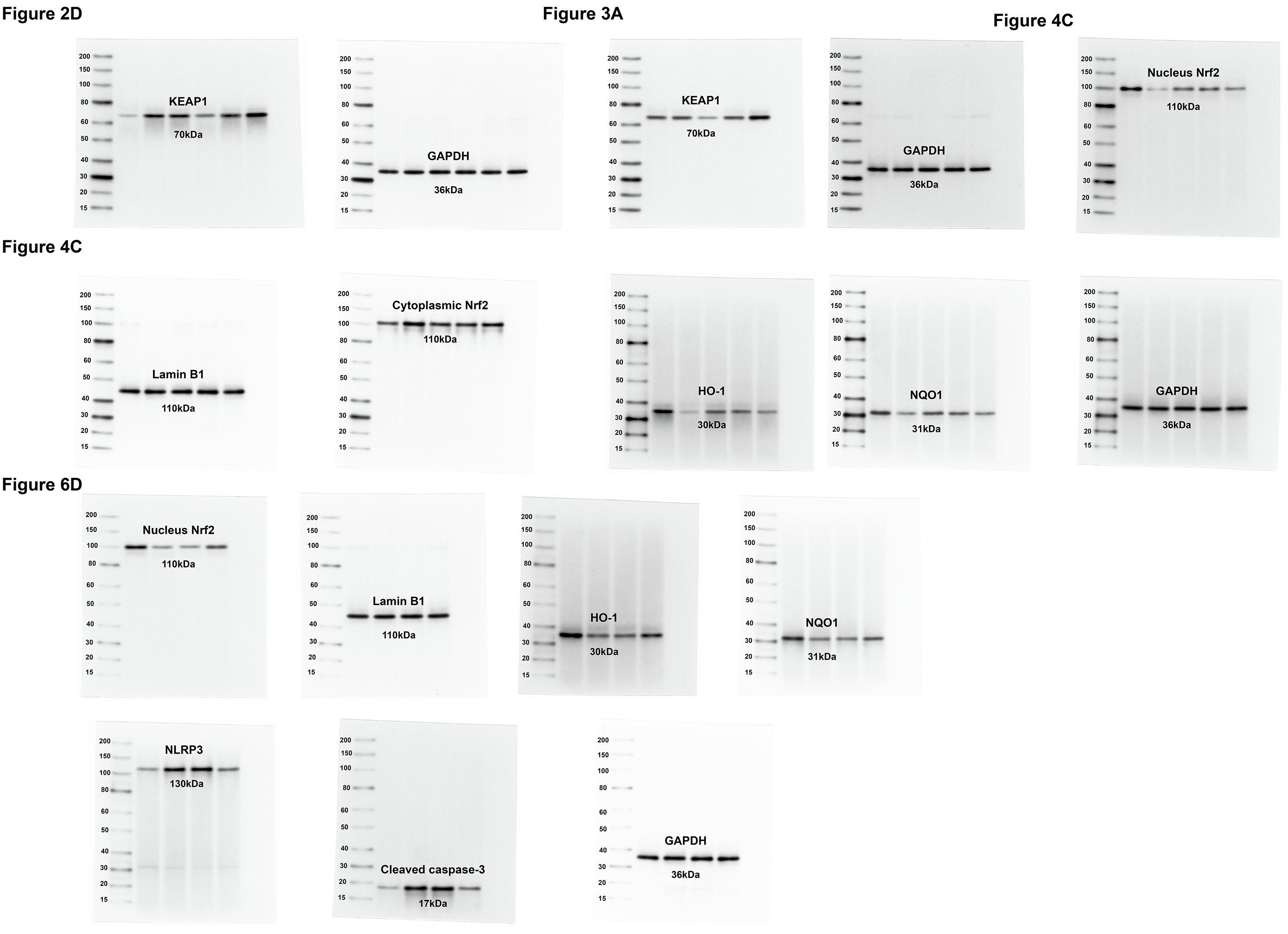

Supplement: Supplementary Figure S1 — All the original and unprocessed protein bands. [file Image1.tif]
